# Binding site identification of G protein-coupled receptors through a 3D Zernike polynomials-based method: application to *C. elegans* olfactory receptors

**DOI:** 10.1007/s10822-021-00434-1

**Published:** 2022-01-01

**Authors:** Lorenzo Di Rienzo, Luca De Flaviis, Giancarlo Ruocco, Viola Folli, Edoardo Milanetti

**Affiliations:** 1 Center for Life Nano- & Neuro-Science, Istituto Italiano di Tecnologia, Viale Regina Elena 291, 00161 Rome, Italy; 2 Department of Physics, Sapienza University, Piazzale Aldo Moro 5, 00185 Rome, Italy

**Keywords:** GPCR, Protein-ligand interaction, Binding site prediction, Protein structure

## Abstract

Studying the binding processes of G protein-coupled receptors (GPCRs) proteins is of particular interest both to better understand the molecular mechanisms that regulate the signaling between the extracellular and intracellular environment and for drug design purposes. In this study, we propose a new computational approach for the identification of the binding site for a specific ligand on a GPCR. The method is based on the Zernike polynomials and performs the ligand-GPCR association through a shape complementarity analysis of the local molecular surfaces. The method is parameter-free and it can distinguish, working on hundreds of experimentally GPCR-ligand complexes, binding pockets from randomly sampled regions on the receptor surface, obtaining an Area Under ROC curve of 0.77. Given its importance both as a model organism and in terms of applications, we thus investigated the olfactory receptors of the *C. elegans*, building a list of associations between 21 GPCRs belonging to its olfactory neurons and a set of possible ligands. Thus, we can not only carry out rapid and efficient screenings of drugs proposed for GPCRs, key targets in many pathologies, but also we laid the groundwork for computational mutagenesis processes, aimed at increasing or decreasing the binding affinity between ligands and receptors.

## Introduction

The identification and characterization of ligand binding sites in proteins is a fundamental step for structure-based drug design [1, 2]. Among all protein families, G protein-coupled receptors (GPCRs) are probably the most popular family of drug targets, accounting for about 34% of all pharmaceuticals approved by the US Food and Drug Administration [3].

GPCR proteins are characterized by a seven-helices trans-membrane domain, that winds through the plasma membrane in a serpentine fashion. Besides, they are composed of an N-terminal domain, which is oriented to face the extracellular matrix and, in some cases, can participate in ligand binding, and a C-terminal domain, which is in the cytosol and is involved in the signaling inside the cell. Moreover, to maintain the serpentine structure, 3 extracellular and 3 intracellular loops are used for connecting the alpha helices trans-membrane regions among them [4]. The extracellular loops can contribute to ligand binding as well [5].

GPCRs play a key function in many biological processes, mediating a large number of cellular responses to external stimuli, such as light, odors, hormones, and growth factors. The communication between the outside and the inside of the cell takes place through the interaction between GPCRs and ligands in the extracellular environment, allowing the protein to bind, in an intracellular environment, with a heterotrimeric G protein [6]. This process is followed by various forms of signal transduction, all these events originated by the interaction of the GPCR with a specific ligand [7].

Therefore, the understanding of the interaction mechanism between small molecules and GPCRs represents a fundamental step for the design of new pharmaceutical compounds [8]. Among all the GPCR-ligand interactions, a relevant part is the recognition mechanisms between odors and their corresponding Odor Receptors (ORs), which represent the largest subfamily within the GPCRs family and are present in all multicellular organisms [9–11]. Indeed, these interactions are fundamental for many biological applications [1]. For instance, the level of expression of ORs in tumoral tissues is noticeably different compared to healthy tissues, and, currently, these receptors are candidates as new targets for diagnosis and therapeutics, but a complete comprehension of their biological role in cancer is still elusive [12]. More specifically, it is still unclear how olfactory receptors (ORs) recognise volatile molecules [13–15], making the design of specific receptors for any given odorant difficult [16]. Di Pizio et al. recently reviewed in detail the role of ORs in physiological and pathological processes [17].

As easily predictable given the importance of GPCR proteins, in recent years many biochemistry and bioinformatics approaches have been developed to study drug-receptor binding [7, 18, 19], and some of them have been even applied for the prediction of their corresponding binding affinity [20, 21]. As for other protein-ligand prediction methods, most of them are based on machine learning methods such as SVM (Support Vector Machine) [22–24] and Neural Network [25, 26], but unfortunately only a limited amount of structural data are available for training these models.

Indeed, since GPCRs are integral membrane proteins containing seven transmembranes (TM) $\alpha$-helices and are characterized by flexible and dynamic structures, it is very difficult to obtain experimental structures based on biochemical and crystallographic experiments [27]. For this reason, computational studies aimed at modeling the three-dimensional structure and, eventually, the association between GPCR and ligand are fundamental to investigate the GPCR-ligand interaction properties [28].

Here we present a new procedure based on a parameter-free computational approach, able to recognize, completely unsupervised, if a region of a GPCR protein can be involved in an interaction with a given ligand.

**Fig. 1:**
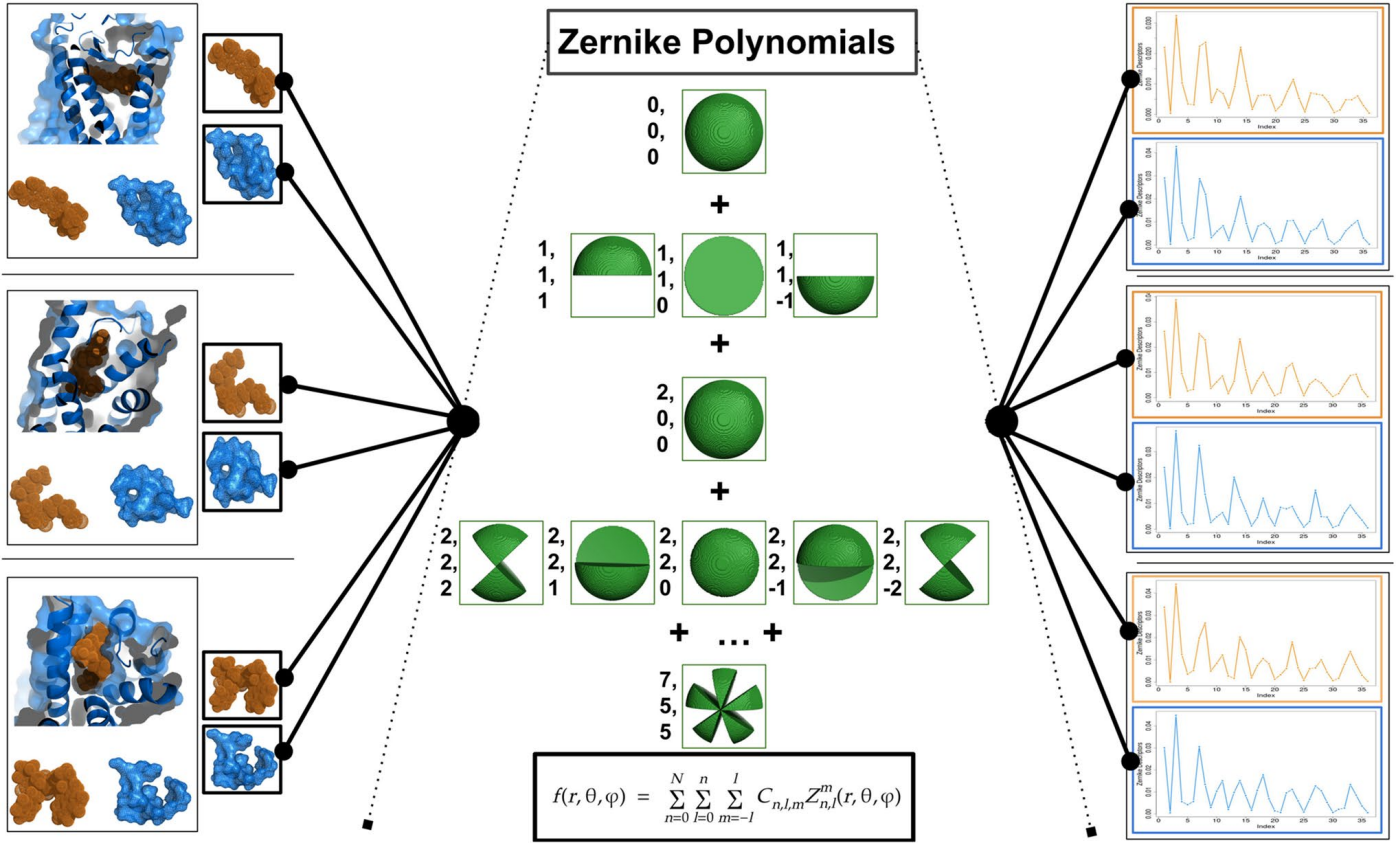
Schematic Representation of the various step in our computational protocol. Starting from experimental structures of GPCR-ligand complexes, we extracted the molecular surfaces of the protein binding regions and the ligands (left panels). These molecular surfaces can be expanded on the basis of 3D Zernike polynomials (central panel). The norm of the coefficients of this expansion constitute a set of rotationally invariant descriptors, summarizing the shape of the extracted surface (right panels). To compute the complementarity between two interacting surfaces, we compute a distance between their corresponding set of Zernike descriptors

The method is based on the 3D formalism of the Zernike polynomials [29, 30] and it can characterize with an ordered set of numerical descriptors the morphological properties of a molecular surface. In particular, we computed the molecular surface [31] of a protein and we extract the portion generated by a specific set of residues (patch). This patch can be represented as a function in the 3d space that can be expanded on the basis of the Zernike polynomials (see Methods, Eq. 3). The coefficients of such expansion are determined by the form of the specific patch analyzed since they represent the weights that each polynomial assumes in the expansion. The Zernike descriptors are obtained by computing the norms of such coefficients, and they are invariant under rotation and translation in 3D space. Therefore, each selected molecular shape is summarized with its corresponding Zernike descriptors. We calculated the Zernike descriptors for the ligand binding site and for the ligand itself, being able to compute their compatibility simply by calculating the distance between the corresponding descriptors (see Figure 1).

This framework allows us to effectively evaluate the compatibility between two molecules because, in principle, two perfectly fitting surfaces are identical and then they share the same Zernike descriptors. Therefore, the distance observed between interacting surfaces (ligand–binding site) is significantly lower than the one observed between non-interacting surfaces (ligand – randomly selected exposed region of the same size of the binding site). In the last years, the Zernike formalism has been widely applied for the study of molecular complementarity [32–38].

Thus, we collected a large structural crystallographic dataset of GPCR-ligand experimental complexes. Computing the shape complementarity employing Zernike descriptors, the difference, in terms of ligand compatibility, between binding sites or randomly surface regions is clearly recognizable.

We thus applied the proposed strategy to investigate the ligand-receptor interaction in the nematode *Caenorhabditis elegans*. This tiny roundworm detects a large class of volatile odorants, making the olfaction its primary sense, and represents a model organism for studying and addressing the biology of olfaction. The olfactory system of *C. elegans* comprises approximately 32 chemosensory neurons and more than 5% of its overall genes are related to sensing chemical cues in the environment [39]. Unlike mammalian olfactory neurons, each expressing just one kind of olfactory receptor, *C. elegans* chemosensory neurons express more than one receptor per cell and each worm’s chemosensory neuron may respond to a large variety of molecules. As a consequence, despite the low number of constituting cells, the olfactory circuit of *C. elegans* recognizes hundreds of molecules at a wide range of concentrations [13, 39, 40].

Studying the olfactory network in *C. elegans* may help in addressing a challenge more demanding in higher brain systems: how does the neural coding strategy of odors actually work? Despite the compact nervous system, the nematode is capable to sense and integrate into reproducible behaviors a multitude of olfactory signals. *C. elegans* provides therefore a unique and easy-to-manage platform to study neural circuits underlying smell.

However, the large co-expression of GPCRs within olfactory neurons makes genetic screens ineffective in linking receptors to odors and only a few GPCRs have been linked to the corresponding ligands [41]. Using the method presented in this paper, we build a comprehensive list of odorant-receptor possible associations in an olfactory neuron of *C. elegans*. However, it is worth noting that this protocol can be applied in analogous types of analysis for every kind of chemosensory receptors.

Specifically, we select 21 among GPCRs expressed on the main olfactory neuron mediating attraction in *C. elegans*, the AWC neuron [42]. AWC neurons are a pair of olfactory neurons critical for chemotaxis to volatile odorants (e.g. attraction to benzaldehyde, butanone, isoamylalcohol, 2,3 pentanedione, and 2,4,5 trimethylthiazole) [13]. Their activation induces local search behavior and promotes turns. We tested these 21 GPCRs against 18 putative ligands we identified in a recent paper using a sequence-based methodology [43]. In particular, sampling the 21 olfactory receptors of *C. elegans*, we computed the shape complementarity between each receptor patch and the proposed ligands. Thus we can evaluate these results with respect to the typical complementary values observed in experimental GPCR-molecule interactions. From this perspective, with an *in-silico* characterization we propose a set of GPCR-ligand interactions in *C. elegans* since our formalism shows that that couple exhibits an exceptionally high shape complementarity.

## Results and discussions

The identification of the binding region responsible for the capture of a chemical compound on a GPCR is a key aspect both to understanding the molecular recognition mechanism and to open the door to new methodologies for drug design and optimization of ligand-GPCR interactions. For example, it is possible to design an *ad-hoc* compound able to compete with the physiological ligand or, employing computational mutagenesis protocols, amino acid substitutions can be carried out to increase (or decrease) protein-ligand binding compatibility.

In this study we developed a new computational procedure, completely based on shape complementarity analysis, able to distinguish the specific interaction occurring between a ligand and its GPCR binding region from the non-specific ones, involving a set of randomly sampled regions on the surface of the protein itself. To this end, we selected a large experimental dataset and analyzed the geometric properties of the molecular surface regions by adopting the Zernike polynomials description, which is a powerful tool in capturing the structural determinants of both protein-protein and protein-ligand recognition [32–36, 38, 44].

After demonstrating that the sensibility of the employed representation is good enough to properly work even when a predicted protein structure is considered (a significant aspect due to the lack of the experimentally resolved GPCRs structure), we applied the computational procedure to investigate the odor-receptor recognition mechanisms of the model organism *C. elegans*. Here, we propose some possible associations between the putative ligands identified in [43] and a set of 21 GPCRs belonging to AWC neuron of *C. elegans*.

### Detection of ligand-specific binding site regions on GPCRs

**Fig. 2:**
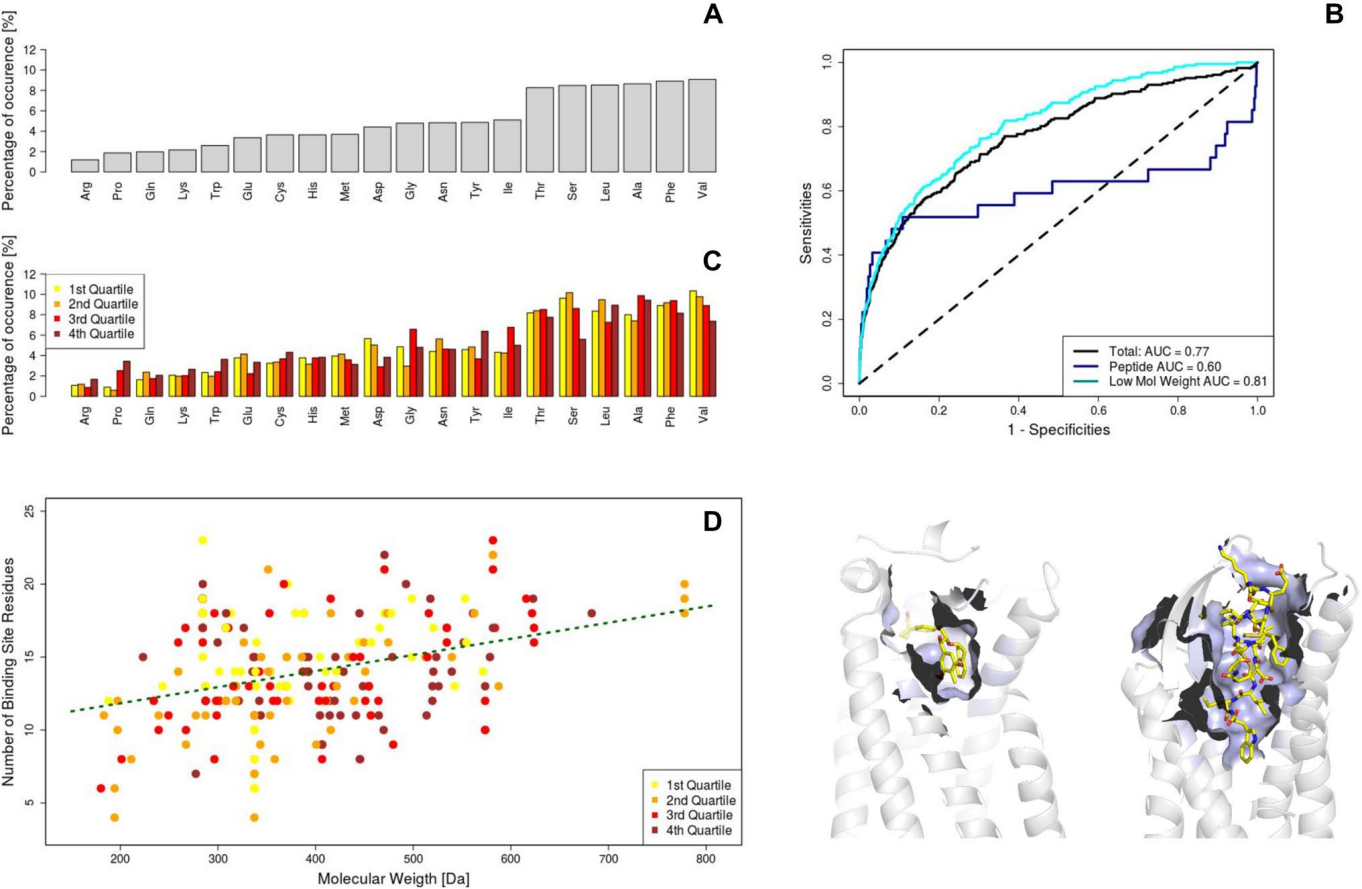
Analysis of the interaction between ligands and GPCRs binding sites: application of the Zernike formalism. **A** The amino acid distribution regarding the amino acids involved in binding. **B** ROC curves obtained using the Zernike Descriptors. The green line (AUC = 0.77) regards all the dataset, the red line (AUC = 0.60) consider only the interaction between GPCR protein and peptide ligand, while the blue line is related to the ligands with a molecular weight lower than 500 Da (AUC = 0.81). The molecular images represent an example of GPCR-small molecule and GPCR-peptide recognition, respectively on the left and the right. **C** The same as in **A**, but the results are grouped according to the membership of each GPCR-ligand complex to one of the quartiles of Zernike accuracy (Cyan = $1{st}$ quartile means high complementarity while Brown = 4st quartile means low complementarity.) **D** The number of Binding site residues as a function of the molecular weight of the ligand. The points are colored according to the membership to one of the quartiles of Zernike accuracy (Cyan = 1st quartile means high complementarity while Brown = 4th quartile means low complementarity.)

We collected a dataset of 287 experimentally solved GPCR-ligand complexes. For each structure, we defined the GPCRs binding sites (BS) as the set of residues whose $C\alpha$ are closer than 6 Åto any atoms of the ligand.

We first analyzed the frequencies of amino acids occurrences in the binding regions, as shown in the barplot in Fig. 2A. Comparing our results with the corresponding ones regarding the binding sites of a systematic dataset of protein-ligand complexes (about 4000), some remarkable differences turn out [45]. Some non-polar residues, such as Ala and Val, increase largely their frequencies, while charged residues (Arg, Lys, Glu, Asp) are very low represented in GPCRs binding sites. Moreover, it can be noted that interactions mediated by Tyr and Trp aromatic residues are less common than in the general case: on the contrary, some polar residues (Thr, Ser) increase their occurrences. Taken together, these results seem to give insights into the peculiar nature of the GPCRs binding sites, often located in a very internal area with limited solvent exposure.

We defined on each GPCR a set of randomly selected surface regions (Binding Site Decoy, BSD), in such a way that these are as comparable as possible with actual BS (See Methods Section). Thus, we calculated the Zernike descriptors of both the BS and the BSD, and the ligand as well. We, therefore, summarized in an ordered vector of numerical values the geometric properties of the protein patches (BS and BSD) and the ligand. Since the shape of two perfectly fitting surfaces is the same, we quantified the complementarities between protein and ligand computing the distance between the corresponding Zernike descriptors, where lower is the distance higher is the complementarity (See Methods section).

This analysis aims to verify if the Zernike description can distinguish, in terms of shape complementarity, specific interactions (ligand-binding site) from non-specific ones (ligand-decoys), analyzing in this way both the performance of the designed method and the role of shape complementarity in receptor-ligand recognition. The main hypothesis of this study is that the binding between ligand and receptor is mainly mediated by the geometric complementarity of the two molecules. In this scenario, since the actual binding sites should achieve a higher shape complementarity with the ligand than other surface regions, the distance between the descriptors of the ligand and the binding site should be lower than the ones involving the decoys.

Collecting the results regarding all the complexes in the dataset, the distribution of the specific interaction Z-score is characterized by values significantly lower than 0, meaning that the formalism on average capture the differences in shape complementarity between specific and non-specific interactions. In particular, the specific interaction Z-scores are characterized by a mean of -1.07 and a standard deviation of 1.18.

In the light of these results, we performed a ROC analysis to quantify the protocol binding site prediction power (Fig. 2B): labeling as positive the actual BS and as negatives the BSD, and using the Zernike distances with the cognate ligands as discriminant, we obtained an area under the curve (AUC) of 0.77, meaning that we satisfactorily spot the correct protein region-ligand associations.

Interestingly, our method shows different performances depending on the kind of ligand bound by the GPCR. Indeed, when only peptidic ligands are considered the performances drastically decrease (AUC = 0.60), while when only small ligands (molecular weight lower than 500 Da) are examined, the computational classification protocol works even better than in general case (AUC = 0.81). Since peptides are usually characterized by a high molecular weight in respect to non-peptidic ligands, a possible interpretation can be that ligand-pocket shape complementarity is sufficient for the identification of pockets suitable for the recruitment of small compounds, while for the recognition of heavier ligands it is also necessary a good chemical-physical interaction [46, 47]. Indeed, as shown in the molecular images in Fig. 2B, the shape complementarity can be caught by Zernike description very effectively when a small molecule is considered, since a small pocket is perfectly built around the ligand. When the molecule recognized by a GPCR is characterized by a high molecular weight ( as the peptides usually are), the binding site that recognizes it is connected to the outside solvent. Likewise, a large portion of the ligand molecular surface is not optimized to bind the protein binding site since it is not in direct contact with the protein. Therefore, when such molecule is expanded on the Zernike basis, the descriptors will represent also the part of the surface that is not complementary to the protein binding site, resulting inevitably in a decrease of protein-ligand shape complementarity.

To verify if certain residues can mediate preferentially shape complementarity with ligands, we thus grouped GPCR-ligand complexes into 4 categories depending on the accuracy the method achieves in detecting the actual binding site. In particular, each GPCR was assigned in the corresponding quartile of the Z-scores distribution, so as the $1{st}$ quartile contains the complexes best classified while the $4{th}$ one incorporates the GPCR-ligand complexes with the worst scores.

In Fig. 2.C we reported the frequencies of amino acids in the binding site separated according to the 4 quartiles. Interestingly, in the GPCRs binding sites characterized by a high shape complementarity are over-expressed some amino acids, for instance Valine (Val) and Serine (Ser), suggesting that such small and flexible residues can effectively adapt themselves to accommodate the ligand.

Moreover, in Fig. 2.D, we showed the number of residues involved in binding sites as a function of the ligand molecular weight. As expected, a linear correlation between two quantities exists (Pearson correlation coefficient of 0.32, p value $< 10^{-5}$). The points in this plot are colored in according to the quartile membership of each molecular complex: even if lighter ligands are slightly better predicted(see Figure 2.B), there are no significant separations between these groups and therefore we can conclude that the description based on Zernike moments, in this range, is not biased by the binding site size (note that in this analysis we not consider peptide ligands).

### Evaluation of ligand flexibility in shape complementarity with GPCRs

**Fig. 3:**
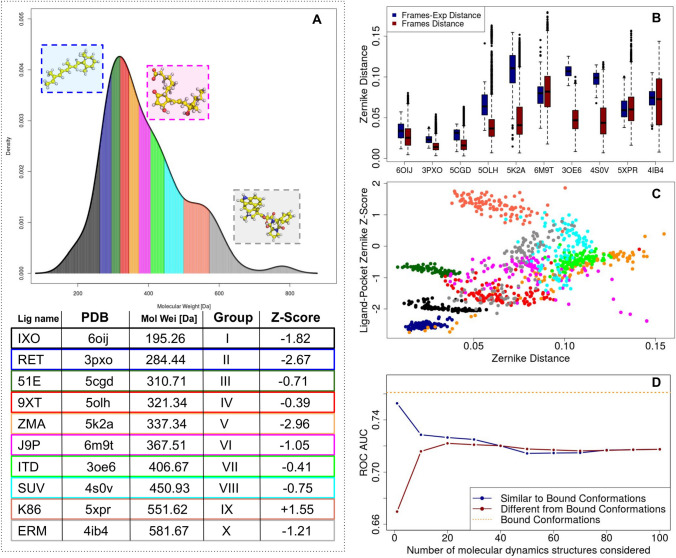
Evaluation of shape complementarity between different ligand conformations and cognate GPCRs. **A** Density distribution of the molecular weights of the ligand in our dataset. The area corresponding to each decile of the distribution is colored with a different color. We selected one small ì-molecule from each decile to study their flexibility with molecular dynamics simulation. In the colored boxes we report the molecular representation of three selected molecules, i.e. RET (PDB code: 3pxo), J9P (PDB code: 6m9t), ERM (PDB code :4ib4). **B** For each ligand selected, we report in blue the Zernike descriptors distances between the experimental ligand conformations and all the molecular dynamics frames, and in red the distances between all the explored frames in the simulation. **C** The Z-score computed for each frame of each molecular dynamics as a function of the distance between each frame with the corresponding ligand bound conformation. **D** The Area Under ROC curve we obtained as a function of the frames considered. In the blue curve, we consider ligand conformations progressively less similar to the bound one. In the red curve, we consider ligand conformations progressively more similar to the bound one. The performance we obtained is better when ligand structures similar to the bound state are considered

The compatibility between a protein binding site and a ligand is dependent on the conformation adopted by the ligand. Bound conformations are usually characterized by a shape complementarity higher than the one observed when unbound states (APO conformation) are investigated. Thus, in this section, we investigated how ligand flexibility impacts the compatibility with the GPCR binding site. In particular, we selected a subset of ligands and we performed a molecular dynamics analysis to establish how their flexibility impacts the compatibility with the protein binding site. We summarized the results of this analysis in Fig. 3.

Looking at the distribution of the molecular weights of the ligands in our dataset, we selected one ligand from each decile. Therefore, we constituted a subset of 10 ligands stratified in terms of molecular weight (see Fig. 3A). To explore the conformational space of each ligand, we extracted 100 different ligand conformations sampling uniformly each ns a 100-ns long molecular dynamics simulation. Finally, we summarized the shape features of each obtained conformation by computing its Zernike descriptors. For each simulation, we computed the Zernike descriptors distances between the experimental ligand conformations and all the molecular dynamics frames (Fig. 3B blue boxplots). Moreover, we computed the distances between all the explored frames in the simulation (Fig. 3B red boxplots). Therefore, the red distributions represent the level of variability of each structure. The blue distribution, the distances between all the possible conformations with the experimental one, is typically higher than the red one. This is because the bound state represents a small region in the configurations space, often not located in a very explored area. This notwithstanding, the overlapping between the 2 distributions means that the molecular dynamics of the ligand explore some conformations similar to the bound state.

We studied as the difference between the unbound states and the bound one, in terms of Zernike descriptors, is correlated with the pocket-ligand specificity (Fig. 3C). In particular, for each frame of the simulations, we calculated the complementarity between the particular ligand conformation and the experimental binding pocket or the decoys. Thus, we normalized with the Z-score as explained in the manuscript, so as the Z-score of the ligand-binding pocket association is a measure of the sensibility of our approach (the more negative the Z-score is, the more correct is the association between ligand structure and the pocket). As evident, exist a correlation between the difference with the bound state and the capability of recognizing the binding pocket (Pearson correlation coefficient = 0.45). It is interesting to note that the points out of the linear trend are mainly belonging to the dynamics of K86(5xpr), a ligand that we can not correctly assign to its pocket even using the experimental conformation (its experimental Z-score is 1.55).

Finally, we calculated the area under the ROC curve obtained when molecular dynamics frames are considered (Fig. 3D). Ordering the frames according to their similarity with the experimental ligand structure, we report in the figure the mean AUC we obtained considering frames from most to least similar (blue curve) or from least to most similar (red curve). The orange dashed line represents the AUC we obtained with experimental conformation, for this specific subset of GPCR-ligand interactions. It has to be noted that when we consider the structure most similar to the experimental one, the mean performance we get (AUC = 0.75) is very similar to the experimental case (0.76), while the mean performance obtained when the ligand structure is least similar to the bound state is significantly lower (0.67). Including a number progressively higher of structures, by construction, the 2 performances tend to become more and more similar until the perfect identity of the last point (when we consider all the structures of the simulations in both cases). Intuitively, as long as the unbound structure is similar to the bound one, we obtain performances very similar to those obtained in such case; indeed, the conformations explored in dynamics are close enough to the bound state to make possible a satisfying performance.

### The application of Zernike formalism to GPCRs models

**Fig. 4:**
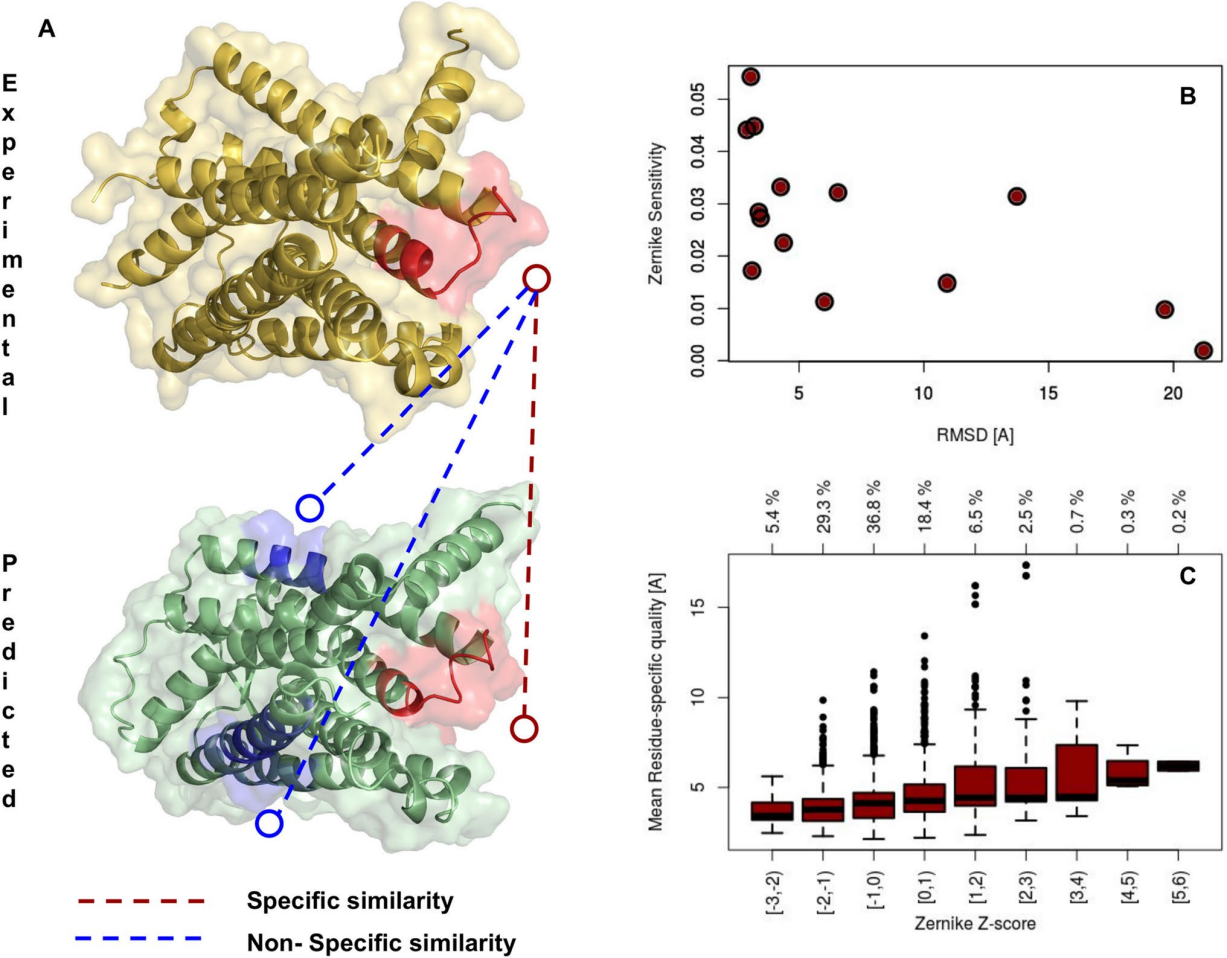
Comparison between experimental and predicted GPCR structures. **A** Molecular representation of an experimental and predicted structure of a GPCR (pdb:41ar). The similarity between the red regions in these 2 proteins, centered on the same residues on both structures, constitutes the *specific similarities* ( see Eq. 1), while the comparisons between unrelated regions, depicted in blue and red in this representation, constitute the *non specific similarities* ( see Eq. 2). **B** The Sensitivity of the Zernike method, defined as $S{ns} - S_{s}$, as a function of the RMSD between experimental and predicted structures. **C** Boxplot representing the Mean L-score of the residues constituting a pocket as a function of the difference between specific and non-specific similarities calculated and normalized on a single pocket. On the top are reported the percentage of pockets in each interval: it results than over 70% has a Z-score lower than 0, highlighting a specific similarity statistically better than non-specific ones

In the previous section, we demonstrated that our approach based on Zernike formalism obtains very good results in binding site identification when applied to experimental GPCR-ligand structures. Unfortunately, the structural details of a very large fraction of GPCRs are still lacking [48, 49], also because of the inherent difficulties of working with membrane proteins.

Therefore, to extend the applicability of this formalism to the whole GPCR family, we need to establish if the shape representation employing Zernike descriptors works properly even when we deal with predictions of GPCR structures provided that, obviously, the results of this study will depend on the quality of the predicted structures.

In light of these considerations, we tested our protocol selecting a subset of the structural dataset and investigating the similarities between predicted and experimentally solved structures. In particular, choosing 14 structures (approximately 5% of the entire dataset) belonging to different classes of GPCRs (according to GPCR-EXP database [50]), we extracted the protein amino acid sequence and we modeled the structure using the I-Tasser web-server [51], excluding from the possible template for homology modeling known structures with too high sequence similarity (all possible templates with sequence identity higher than 25% have been excluded).

We thus compare the native experimental structures and the predicted ones. For each GPCR, we defined for both the experimental and predicted structures a set of surface regions, basically centering on each residue a spherical patch with a radius of 9 Å. We computed the Zernike descriptors of all these patches. It is hence possible to define on one hand the *specific similarities*, the distances between the corresponding patches on the experimental and modeled structure (those centered on the same residues), and on the other hand the *non-specific similarities*, the distances between all the couples of non-corresponding patches (centered on different residues), as illustrated in Fig. 4.A. If the modeling works properly, the mean of the specific similarities has to be lower than the mean of the non-specific similarities.

Therefore, for each protein sampled with i = 1,…,N patches, it holds

1 $$S_s= \frac{1}{N} \sum _{i=1}^N S_{s,i} = \frac{1}{N} \sum _{i = 1}^N dist(E_i, M_i)$$

2 $$S_{ns}= \frac{2}{N(N-1)} \sum _{i=1}^N \sum _{j>1}^N S_{s,ij} = \sum _{i = 1}^N \sum _{j>1}^N dist(E_i, M_j)$$

where $E_i$ and $M_i$ are the Zernike descriptors of the i-th patch relative to the Experimental or Modeled structures, respectively. $S_s$ and $S_{ns}$ thus represent the specific and non-specific similarity relative to the examined protein. We therefore defined the Zernike Sensitivity as $S_Z = S_{ns} - S_s$, meaning that when $S_Z > 0$ our representation preferentially associate corresponding regions.

In Fig. 4.B, we reported for each protein the Zernike Sensitivity as a function of the Root Mean Square Deviation (RMSD) between the modeled and the experimental structure (both the superposition and the calculation was based on $C\alpha$ atoms). When the model has a good quality (low RMSD) the Zernike protocol performs well in recognizing the same region in the 2 structures (high Zernike sensitivity) (Pearson Correlation Coefficient R = -0.65, p-value = 0.01).

Moreover, beyond the global quality of a model and the corresponding Zernike accuracy, it is interesting to study even the local goodness of the predicted structure. Indeed, the I-Tasser web server returns an estimation of the exactness of the single residue position prediction (L-score [52]).

Calculating the mean L-score of each region previously defined we basically defined the confidence of this region prediction. For each experimental-modeled pair of structures, we normalized the distribution of specific similarities and non-specific similarities with the Z-score, similarly to what was done above. In this way, when a region exhibits a low similarity Z-score it means that the specific similarity is better than the typical non-specific one.

In conclusion, we compared the similarity Z-scores with the mean L-score of the region and we reported the results in the boxplot shown in Fig.4.C: as expected, when the local predicted quality of the model is good (low L-score) even the Zernike formalism can recognize better the similarity between predicted and experimental structure.

In this section, we demonstrated that both from the global or local point of view, as long as the protein modeling reaches good results the Zernike description grasp the similarity of the regions and can be therefore applied, with good approximation, on predicted structures.

### Olfactory receptors: the case of *Caenorhabditis elegans*

In this section we focused on the olfactory receptors of the model organism *C. elegans* [42].

**Table 1:** The putative GPCRs ligands selected in this analysis

PDB entry	Ligand	Pubchem CID [53]	X-ray resol [Å]
5U09	Taranabant	11226090	2.6
4N6H	Naltrindole	5497186	1.8
5NDD	AZ8838	126961334	2.8
5ZBH	BMS-193885	9960164	3
4IB4	Ergotamine	8223	2.7
5G53	N-Ethyl-5’-Carboxamido Adenosine	8804901	3.4
5L7D	Cholesterol	5997	3.2
4DJH	JDTic	9956146	2.9
5TGZ	AM6538	46912833	2.8
1U19	Retinal	638015	2.2
2YDO	Adenosine	60961	3.0
4Z34	ONO9780307	252166790	3.0
4BVN	Cyanopindolol	46937143	2.1
4JSP	ATPgammaS	44123300	3.3
3EML	ZM241385	176407	2.6
3UON	Quinuclidinyl benzilate	688566	3.0
2RH1	(S)-Carazolol	13023332	2.4
4IAQ	Dihydroergotamine	10531	2.8

The first column reports the PDB code of the experimental structure where each ligand has been found in interaction with a GPCR, and from which is taken its 3D structure

In a recent work [43], we have designed a new sequence-based strategy to determine the association between each OR and a set of new possible volatile compounds, based on the hypothesis that similar compounds bind similar proteins [54]. In that work, we produced a list of possible ligands for *C. elegans* GPCRs, reported in Table 1, where compounds have been considered if they have been found experimentally in complex with a GPCR at least once.

**Fig. 5:**
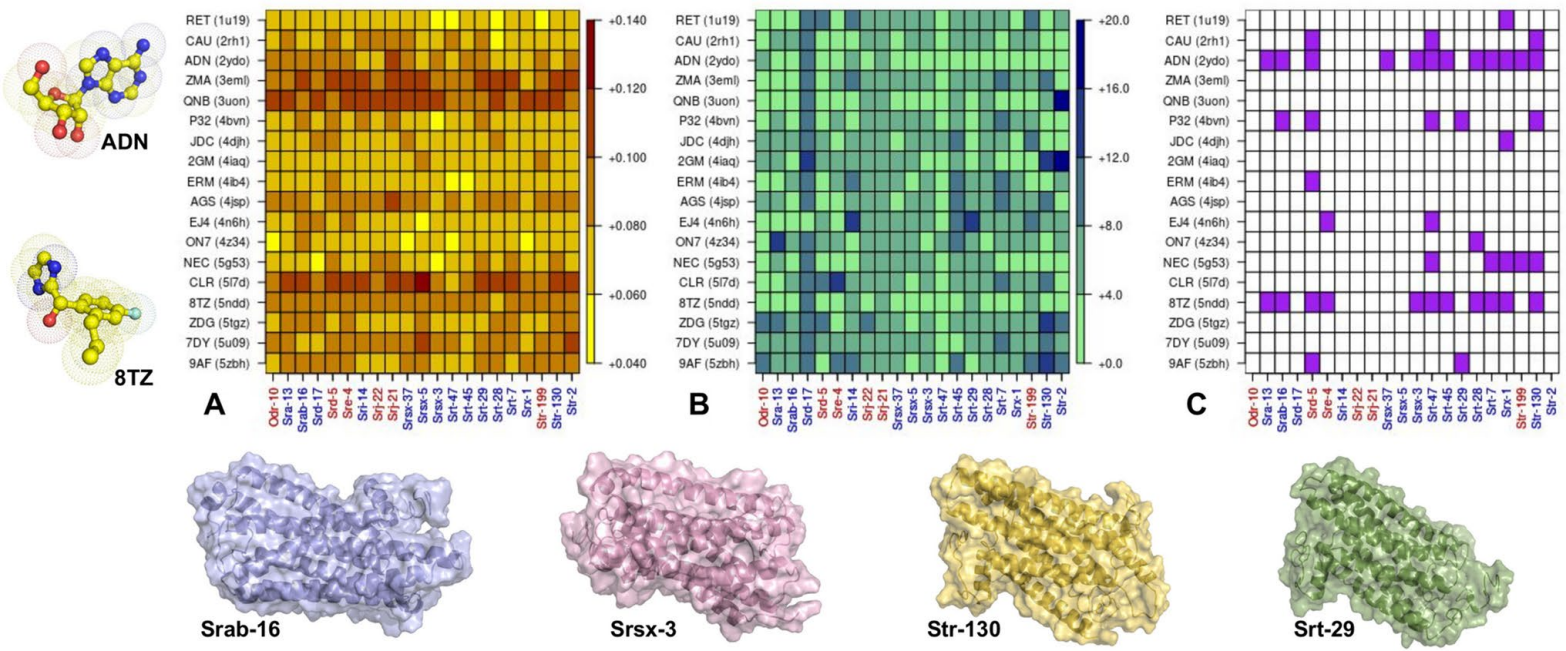
Summary of the associations between ligands and *C. elegans* GPCRs. **A** The distances, computed in terms of their Zernike descriptors, between each putative ligand and the most suitable pocket on each protein structure. It is important to note that when the distance is low (yellow pixel) the complementarity is high. **B** For each GPCR, the mean L-score of the residues constituting the pocket characterized by the best complementarity with each ligand is reported. **C** The hypothesized associations between protein and ligands are colored. On x-axis of A), **B** and, **C** the GPCRs highlighted in blue print refers to models with a C-score higher than − 1.5, those highlighted in red print to models with a C-score lower than − 1.5. In addition we reported the molecular representation of the two ligands characterized by the highest number of possible GPCR associations and four GPCR proteins

To structurally predict possible associations between ligands and receptors, in this section we assessed the shape complementarity observed between each protein and each ligand.

Since the structural details of these proteins are still not available, we modeled them using GPCR I-Tasser web-server. In the previous section, we have shown that when the model is good enough our approach can accurately summarize local shape information. Here, since we can not make the comparison with the experimental structures, we collected for each model the cognate C-score, a confidence score for estimating the quality of the predicted model, as returned by the web-server. As stated here [55], since the C-score highly correlates with the RMSD, when it is higher than − 1.5 the model can be considered satisfactory. In panels A, B,C of Fig. 5, the GPCRs related to models with a C-score higher than − 1.5 are highlighted in blue print while those with a C-score lower than − 1.5 are highlighted in red print.

Thus, we extracted from each GPCR a set of possible ligand binding sites, simply centering on a residue and selecting all the residues closer than 9 Åto it. Selecting the surface points generated by these residues, we defined the patches and computed the Zernike descriptors of each of them. Moreover, we described with the corresponding Zernike descriptors also all the 18 chosen ligands.

We then computed the complementarities between ligands and GPCRs regions, labeling each ligand-GPCR pair with the higher shape (lower distance) compatibility between the compound and one of the protein pockets (Fig. 5A). At the same time, we reported in Fig. 5B the local mean L-score of the selected protein pockets, characterized by the best complementarity value of the ligand-GPCR pair.

We established an association between a ligand and a GPCR if the Zernike distance is lower than 0.09, the upper bound of the 5% lower interval of the distribution of distances between random surface regions and ligands in the structural dataset. This means that an association is formed when there is a probability lower than 5% that the observed shape complementarity belongs to the distributions of “non-associations”.

Moreover, to further control the goodness of the ligand-protein pairing, we required also that the selected binding region exhibits a good mean L-score, belonging to the first quartile of L-scores considering all the protein regions defined in our modeled GPCRs.

The proposed interactions are depicted in Fig. 5C, where a colored spot is placed when a ligand-protein association is hypothesized. It is interesting to note that 2 ligands, AZ8838 (8TZ) and Adenosine (ADN), show a high number of possible associations: this behavior can be due to the small size of these compounds (molecular weight of 219 and 254 Da respectively), that make them compatible, in terms of shape complementarity, with a larger set of protein binding pockets.

## Conclusions

Given their centrality in several biological processes and their importance as a drug target, GPCR proteins have been largely studied to understand the mechanism of interaction with ligands. Here, using a method based on Zernike polynomials on a large dataset of ligand-GPCR X-ray structures, we quantitatively demonstrated that the ligand recognition is mediated by a high shape complementarity with the protein binding sites. We thus assessed the impact of ligand flexibility, studied with molecular dynamics simulation, on the sensibility of our method: we showed that as long as the conformational exploration can reach molecular conformation similar to the experimental ones, our formalism reaches performances in line with those obtained using experimental data. Moreover, the reliability of the Zernike formalism is confirmed when predicted protein structures are used, and this aspect is particularly relevant considering the inherent difficulty in obtaining experimental GPCR structures.

Based on these results, we designed a new fully computational procedure for the GPCR-ligand association and binding sites prediction. Indeed, working on some proteins of the olfactory network in *C.elegans* and on a set of possible ligands, we can hypothesize an association if the complementarity between a small molecule and a confidently predicted protein pocket is exceptionally high.

Undoubtedly, other elements characterizing ligand-pocket compatibility have to be included in the evaluation to improve the method performance. The expansion in the Zernike polynomials can be used also in such a direction, since any Physico-chemical property, such as electrostatic potential or hydrophobicity profile computed on the molecular surface, can be represented with Zernike descriptors [33, 38, 56].

From this point of view, our results can represent a promising step toward the elusive and important goal of predicting GPCRs specificity. The development of reliable and accurate computational methods for GPCR-ligand association can have numerous applications, from driving the design of a drug able to compete for binding in a given pocket to the evaluation of which amino acid substitutions can disrupt a molecular binding.

## Materials and methods

### Datasets

For the study of shape complementarity between small molecule and the corresponding binding region of the GPCR, we used GPCR-EXP database [50], selecting only GPCR-ligand experimentally solved complexes. Among all the complexes available, we have considered the complexes experimentally solved in X-ray crystallography. We have obtained a total of 287 structures and we called it “GPCR-Lig dataset”.

In order to study the molecular mechanisms of *C. elegans* odor recognition via receptor-small molecule interaction, we have selected a set of 21 GPCR genes, 20 of them encoding GPCRs expressed on AWC neurons: sra-13, srab-16, srd-5, srd-17, sre-4, sri-14, srj-21, srj-22, srsx-3, srsx-5, srsx-37, srt-7, srt-28, srt-29, srt-45, srt-47, srx-1, str-2, str-130, str-199 [57]. For this study we include also odr-10, a gene expressed on AWA neuron [43] because, together with sri-14, odr-10 also senses diacetyl (low concentrations versus high concentrations) and it is one of the few GPCRs linked to a specific target molecule [58, 59].

Unfortunately, since any experimental information about their structure is available, a bioinformatic prediction must be performed. We use the GPCR-I-TASSER [51] server protocol to do it and it returns the ten closest X-ray resolved structures to the predicted one. Almost every X-ray resolved structure has at least a “specific ligand” in its template, where by “specific ligand” we assume that this ligand has a pharmaco-biological significance (for example an inhibitor or an activator). We exclude templates with ligands with unclear significance or useful to our analysis (e.g. cofactors, metals, solvents etc). With these criteria, a collection of more than 20 resolved protein templates is thus retrived. We clear each PDB file in order to have a clean “protein-ligand only” file, ready to be submitted to our algorithms.

### Binding site definition

Given each GPCR-ligand complex the binding region was defined by calculating the distance between all C-alpha atoms of the protein and all the atoms of the small molecule. We consider a residue belonging to the real Binding Site (called BS) if it has at least one C-alpha atom with a distance less than 6 A with at least one ligand atom.

For the purpose of comparison, we have adopted the following procedure for the definition of Binsing Site Decoy (called BSD):

- The aim is to define a set of randomly selected BSD on the receptor surface. To this end, we define the BSD in such a way that the number of residue that make up the BSD to be on average as close as possible to the number of BS residue.
- We center a “probe sphere” on each C-alpha of any receptor and we define for each residue a BSD which is dependent on the radius R of the sphere.
- For each residue Ri, the BSD is composed of the residues that have a distance between their C-alpha and the C-alpha of the residue Ri less than R.
- The purpose of this procedure is to vary R in order to find the optimal value in terms of patch size. As R increases, the number of residues increases and the patch size tends to increase. The procedure starts with a radius of 6 A and increasing its dimension by 1 A each iteration step, until a maximum value of 20 A.
- Given a GPCR, we define the BSDs considering the radius R which provides (on average) the number of residues belonging to the BSDs closest to the number of residues belonging to the BS. Therefore, each GPCR has the specific patch decoy size according to the size of the real binding region.

### Surface patch analysis with Zernike descriptors

For each receptor and its cognate ligand, we calculate separately the molecular surface using DMS software [31]. We extracted the portion of protein surface regarding selected residues. Then, with a voxelization procedure, we represent the protein patch and the ligand as 3D functions.

These 3D functions can be expanded in the basis of the 3D Zernike Polynomials [30, 36, 44]: the norm of the expansion coefficients constitutes a ordered set of descriptors that compactly characterize the geometrical shape of the molecular surface.

Indeed, a function $f(r, \theta , \phi )$ can be represented as:

3 $$f(r,\theta ,\phi ) = \sum _{n=0}^{\infty } \sum _{l=0}^{n} \sum _{m=-l}^{l} C_{nlm} Z_{nl}^{m}(r,\theta ,\phi )$$

where $Z_{nl}^{m}$ are the 3D Zernike polynomials, while the coefficients $C_{nlm}$ are defined as Zernike moments.

Zernike polynomials can be written as:

4 $$Z_{nl}^{m}(r,\theta ,\phi ) = R_{nl}(r)Y_l^m(\theta , \phi )$$

where the Y functions are complex spherical harmonics. These functions depend on both the angular variable $\theta$ and $\phi$, while the dependence on the radius variable, r, is enclosed in the function R, which is given by the following expression:

5 $$R_{nl}(r) = \sum _{k=0}^{\frac{(n-l)}{2}} N_{nlk}r^{n-2k}$$

where N is a normalization factor.

Therefore, the 3D Zernike moments of molecular surface (described by a function $f(r,\theta , \phi )$) are defined as the coefficients of the expansion defined in eq 3:

6 $$C_{nlm} = \int _{\mid r \mid \le 1} f({\mathbf {r}}) \overline{Z_{nl}^{m}(r,\theta ,\phi )} d{\mathbf {r}}$$

where ${\overline{Z}}$ is the polynomial complex conjugate.

Their rotation invariant norms constitute the Zernike descriptors:

7 $$D_{nl} = \mid \mid C_{nlm} \mid \mid = \sqrt{\sum _{m=-l}^{l} (C_{nlm})^2}.$$

The detail of the Zernike formalism can be modulated modifying the order of the expansion n. In our implementation, we set the maximum order of expansion was to 10, giving a total of 36 invariants for each surface studied.

Represented as an ordered set of 36 numbers, the shape complementarity between 2 surfaces can be easily evaluated [32, 33, 38]. Since the shape of 2 perfectly fitting surface is exactly the same, they share very similar descriptors. Indeed, using the manhattan distance ($D(\mathbf{X }, \mathbf{Y })= \sum _i \mid X_i - Y_i \mid$), when 2 surfaces have a low distance between them, they are characterized by a similar shape and therefore they are suitable for binding.

For each GPCR-ligand complex, we normalized the complementarity with the Z-score, as following:

8 $$Z_i = \frac{x_i - \mu (x)}{\sigma (x)}$$

where the subscript *i* refers to the $i{\text{-}}th$ patch considered, $x_i$ is the Manhattan distance between its Zernike descriptors and the ones of the ligand, $\mu$ and $\sigma$ represent the mean and the standard deviation of all the patch-ligand distances, respectively.

### Molecular dynamics simulations

All simulations were performed using Gromacs 2020.6 [60]. Parameters of the systems were built using Swiss-Param web server [61]. Molecules was placed in a cubic simulative box, with periodic boundary conditions, filled with TIP3P water molecules [62]. All the molecules were simulated for 100 ns, using an integration step of 1 fs and saving molecules conformations each ns.

## Data Availability

All the GPCR-ligand structures can be freely downloaded from the Protein Data Bank (PDB) (https://www.rcsb.org/) [63]. The protein surface was calculated using with standard parameters dms software [31]. The protein structures analysis and the voxelization were performed using *in-house* R scripts, relying mainly on bio3d [64] and geometry [65] packages. The 3D Zernike descriptors calculation was done using the python code described in [66]. Code is available upon request.

## References

[1] Chan HS (2018). Exploring a new ligand binding site of g protein-coupled receptors. Chem Sci.

[2] Couvineau A, Tan Y-V, Ceraudo E, Laburthe M (2013) Strategies for studying the ligand binding site of gpcrs: photoaffinity labeling of the vpac1 receptor, a prototype of class b gpcrs. In Methods in enzymology. Elsevier, vol 520, 219–237 10.1016/B978-0-12-391861-1.00010-1 23332702

[3] Hauser AS (2018). Pharmacogenomics of gpcr drug targets. Cell.

[4] Rosenbaum DM, Rasmussen SG, Kobilka BK (2009). The structure and function of g-protein-coupled receptors. Nature.

[5] Wheatley M (2012). Lifting the lid on gpcrs: the role of extracellular loops. Br J Pharmacol.

[6] Weis WI, Kobilka BK (2018). The molecular basis of g protein-coupled receptor activation. Annu Rev Biochem.

[7] Seo S (2018). Prediction of gpcr-ligand binding using machine learning algorithms. Comput Math Methods Med.

[8] Gong J (2019). Understanding membrane protein drug targets in computational perspective. Curr Drug Targ.

[9] Buck L, Axel R (1991). A novel multigene family may encode odorant receptors: a molecular basis for odor recognition. Cell.

[10] Niimura Y, Matsui A, Touhara K (2014). Extreme expansion of the olfactory receptor gene repertoire in African elephants and evolutionary dynamics of orthologous gene groups in 13 placental mammals. Genome Res.

[11] de March CA, Kim S-K, Antonczak S, Goddard WA, Golebiowski J (2015). G protein-coupled odorant receptors: from sequence to structure. Protein Sci.

[12] Launay G, Sanz G, Pajot-Augy E, Gibrat J-F (2012). Modeling of mammalian olfactory receptors and docking of odorants. Biophys Rev.

[13] Bargmann CI, Hartwieg E, Horvitz HR (1993). Odorant-selective genes and neurons mediate olfaction in *C. elegans*. Cell.

[14] Jiang Y (2015). Molecular profiling of activated olfactory neurons identifies odorant receptors for odors in vivo. Nat Neurosci.

[15] Dewan A (2018). Single olfactory receptors set odor detection thresholds. Nat Commun.

[16] Teşileanu T, Cocco S, Monasson R, Balasubramanian V (2019). Adaptation of olfactory receptor abundances for efficient coding. Elife.

[17] Di Pizio A, Behrens M, Krautwurst D (2019). Beyond the flavour: the potential druggability of chemosensory g protein-coupled receptors. Int J Mol Sci.

[18] Sandal M (2013). Gomodo: a gpcrs online modeling and docking webserver. PLoS ONE.

[19] Vasile S et al (2018) Characterization of ligand binding to gpcrs through computational methods. In Computational methods for GPCR drug discovery. Springer, Berlin, vol 23–44 10.1007/978-1-4939-7465-8_2 29188557

[20] Glaab E (2016). Building a virtual ligand screening pipeline using free software: a survey. Briefings Bioinform.

[21] Liu Q, Kwoh CK, Li J (2013) Binding affinity prediction for protein-ligand complexes based on $$\beta$$ contacts and b factor. J Chem Inf Model 53:3076–3085 10.1021/ci400450h 24191692

[22] Geppert H, Humrich J, Stumpfe D, Gärtner T, Bajorath J (2009). Ligand prediction from protein sequence and small molecule information using support vector machines and fingerprint descriptors. J Chem Inf Model.

[23] Iacucci E, Ojeda F, De Moor B, Moreau Y (2011). Predicting receptor-ligand pairs through kernel learning. BMC Bioinform.

[24] Jacob L, Vert J-P (2008). Protein-ligand interaction prediction: an improved chemogenomics approach. Bioinformatics.

[25] Givehchi A, Schneider G (2005). Multi-space classification for predicting gpcr-ligands. Mol Divers.

[26] Cheng F, Zhou Y, Li W, Liu G, Tang Y (2012). Prediction of chemical-protein interactions network with weighted network-based inference method. PLoS ONE.

[27] Zhao Q, Wu B-L (2012). Ice breaking in gpcr structural biology. Acta Pharmacol Sin.

[28] Chan WK (2015). Glass: a comprehensive database for experimentally validated gpcr-ligand associations. Bioinformatics.

[29] Canterakis N (1999) 3d zernike moments and zernike affine invariants for 3d image analysis and recognition. In: In 11th Scandinavian conference on image analysis, Citeseer

[30] Novotni M, Klein R (2004). Shape retrieval using 3d zernike descriptors. Comput-Aided Des.

[31] Richards FM (1977). Areas, volumes, packing, and protein structure. Annu Rev Biophys Bioeng.

[32] Venkatraman V, Yang YD, Sael L, Kihara D (2009). Protein-protein docking using region-based 3d zernike descriptors. BMC Bioinform.

[33] Di Rienzo L, Milanetti E, Alba J, D’Abramo M (2020). Quantitative characterization of binding pockets and binding complementarity by means of zernike descriptors. J Chem Inf Model.

[34] Sandomenico A (2021). Insights into the interaction mechanism of dtp3 with mkk7 by using std-nmr and computational approaches. Biomedicines.

[35] Di Rienzo L (2020). A novel strategy for molecular interfaces optimization: the case of ferritin-transferrin receptor interaction. Comput Struct Biotechnol J.

[36] Venkatraman V, Sael L, Kihara D (2009). Potential for protein surface shape analysis using spherical harmonics and 3d zernike descriptors. Cell Biochem Biophys.

[37] Alba J, Rienzo LD, Milanetti E, Acuto O, D’Abramo M (2020). Molecular dynamics simulations reveal canonical conformations in different pmhc/tcr interactions. Cells.

[38] Daberdaku S, Ferrari C (2018). Exploring the potential of 3d zernike descriptors and svm for protein-protein interface prediction. BMC Bioinform.

[39] Bargmann CI (2006) Chemosensation in *C. elegans*. In: WormBook: the online review of *C. elegans* biology [Internet] WormBook 10.1895/wormbook.1.123.1 PMC4781564 18050433

[40] Troemel ER, Chou JH, Dwyer ND, Colbert HA, Bargmann CI (1995). Divergent seven transmembrane receptors are candidate chemosensory receptors in *C. elegans*. Cell.

[41] Bastiani C, Mendel J (2006) Heterotrimeric g proteins in *C. elegans*. WormBook: the online review of *C. elegans* biology [Internet] 10.1895/wormbook.1.75.1 PMC4781550 18050432

[42] White JG, Southgate E, Thomson JN, Brenner S (1986). The structure of the nervous system of the nematode *Caenorhabditis elegans*. Philos Trans R Soc Lond B Biol Sci.

[43] Milanetti E (2019). Investigation of the binding between olfactory receptors and odorant molecules in *C. elegans* organism. Biophys Chem.

[44] Di Rienzo L, Milanetti E, Lepore R, Olimpieri PP, Tramontano A (2017). Superposition-free comparison and clustering of antibody binding sites: implications for the prediction of the nature of their antigen. Sci Rep.

[45] Gallina AM, Bork P, Bordo D (2014). Structural analysis of protein-ligand interactions: the binding of endogenous compounds and of synthetic drugs. J Mol Recogn.

[46] Dagliyan O, Proctor EA, D’Auria KM, Ding F, Dokholyan NV (2011). Structural and dynamic determinants of protein-peptide recognition. Structure.

[47] Kumar A, Zhang KY (2018). Advances in the development of shape similarity methods and their application in drug discovery. Front Chem.

[48] Grisshammer R (2013). Why we need many more g protein-coupled receptor structures. Expert Rev Proteomics.

[49] Lacapere J-J, Pebay-Peyroula E, Neumann J-M, Etchebest C (2007). Determining membrane protein structures: still a challenge!. Trends Biochem Sci.

[50] Chan WK, Zhang Y (2020). Virtual screening of human class-a gpcrs using ligand profiles built on multiple ligand-receptor interactions. J Mol Biol.

[51] Zhang J, Yang J, Jang R, Zhang Y (2015). Gpcr-i-tasser: a hybrid approach to g protein-coupled receptor structure modeling and the application to the human genome. Structure.

[52] Yang J, Wang Y, Zhang Y (2016). Resq: an approach to unified estimation of b-factor and residue-specific error in protein structure prediction. J Mol Biol.

[53] Kim S (2019). Pubchem 2019 update: improved access to chemical data. Nucleic Acids Res.

[54] Liu H, Sun J, Guan J, Zheng J, Zhou S (2015). Improving compound-protein interaction prediction by building up highly credible negative samples. Bioinformatics.

[55] Zhang Y (2008). I-tasser server for protein 3d structure prediction. BMC Bioinform.

[56] Sael L, La D, Li B, Rustamov R, Kihara D (2008). Rapid comparison of properties on protein surface. Proteins: Struct Funct Bioinform.

[57] Vidal B (2018). An atlas of *Caenorhabditis elegans* chemoreceptor expression. PLoS Biol.

[58] Sengupta P, Chou JH, Bargmann CI (1996). odr-10 encodes a seven transmembrane domain olfactory receptor required for responses to the odorant diacetyl. Cell.

[59] Taniguchi G, Uozumi T, Kiriyama K, Kamizaki T, Hirotsu T (2014). Screening of odor-receptor pairs in *Caenorhabditis elegans* reveals different receptors for high and low odor concentrations. Sci Signal.

[60] Spoel DVD (2005). GROMACS: Fast, flexible, and free. J Comput Chem.

[61] Zoete V, Cuendet MA, Grosdidier A, Michielin O (2011). Swissparam: a fast force field generation tool for small organic molecules. J Comput Chem.

[62] Jorgensen WL, Chandrasekhar J, Madura JD, Impey RW, Klein ML (1983). Comparison of simple potential functions for simulating liquid water. J Chem Phys.

[63] Berman HM (2000). The protein data bank. Nucleic Acids Res.

[64] Grant BJ, Rodrigues AP, ElSawy KM, McCammon JA, Caves LS (2006). Bio3d: an r package for the comparative analysis of protein structures. Bioinformatics.

[65] Habel K, Grasman R, Gramacy RB, Mozharovskyi P, Sterratt DC (2019) geometry: mesh generation and surface tessellation. R package version 0.4.1

[66] Grandison S, Roberts C, Morris RJ (2009). The application of 3d zernike moments for the description of “model-free” molecular structure, functional motion, and structural reliability. J Comput Biol.

